# Post-pancreatectomy acute pancreatitis and pancreatic fistula after pancreatoduodenectomy: two distinct but potentially correlated clinical entities

**DOI:** 10.1093/bjsopen/zrae107

**Published:** 2024-09-26

**Authors:** Giuseppe Quero, Claudio Fiorillo, Chiara Lucinato, Flavia Taglioni, Vito Laterza, Edoardo Panza, Giuseppe Massimiani, Teresa Mezza, Roberta Menghi, Ludovica Di Cesare, Beatrice Biffoni, Davide De Sio, Fausto Rosa, Vincenzo Tondolo, Sergio Alfieri

**Affiliations:** Gemelli Pancreatic Center, CRMPG (Advanced Pancreatic Research Center), Fondazione Policlinico Universitario ‘Agostino Gemelli’ IRCCS, Rome, Italy; Università Cattolica del Sacro Cuore di Roma, Rome, Italy; Gemelli Pancreatic Center, CRMPG (Advanced Pancreatic Research Center), Fondazione Policlinico Universitario ‘Agostino Gemelli’ IRCCS, Rome, Italy; Gemelli Pancreatic Center, CRMPG (Advanced Pancreatic Research Center), Fondazione Policlinico Universitario ‘Agostino Gemelli’ IRCCS, Rome, Italy; Gemelli Pancreatic Center, CRMPG (Advanced Pancreatic Research Center), Fondazione Policlinico Universitario ‘Agostino Gemelli’ IRCCS, Rome, Italy; Gemelli Pancreatic Center, CRMPG (Advanced Pancreatic Research Center), Fondazione Policlinico Universitario ‘Agostino Gemelli’ IRCCS, Rome, Italy; Gemelli Pancreatic Center, CRMPG (Advanced Pancreatic Research Center), Fondazione Policlinico Universitario ‘Agostino Gemelli’ IRCCS, Rome, Italy; Gemelli Pancreatic Center, CRMPG (Advanced Pancreatic Research Center), Fondazione Policlinico Universitario ‘Agostino Gemelli’ IRCCS, Rome, Italy; Università Cattolica del Sacro Cuore di Roma, Rome, Italy; Pancreas Unit, CEMAD Centro Malattie dell'Apparato Digerente, Medicina Interna e Gastroenterologia, Fondazione Policlinico Universitario ‘Agostino Gemelli’ IRCCS, Rome, Italy; Gemelli Pancreatic Center, CRMPG (Advanced Pancreatic Research Center), Fondazione Policlinico Universitario ‘Agostino Gemelli’ IRCCS, Rome, Italy; Università Cattolica del Sacro Cuore di Roma, Rome, Italy; Gemelli Pancreatic Center, CRMPG (Advanced Pancreatic Research Center), Fondazione Policlinico Universitario ‘Agostino Gemelli’ IRCCS, Rome, Italy; Gemelli Pancreatic Center, CRMPG (Advanced Pancreatic Research Center), Fondazione Policlinico Universitario ‘Agostino Gemelli’ IRCCS, Rome, Italy; Gemelli Pancreatic Center, CRMPG (Advanced Pancreatic Research Center), Fondazione Policlinico Universitario ‘Agostino Gemelli’ IRCCS, Rome, Italy; Gemelli Pancreatic Center, CRMPG (Advanced Pancreatic Research Center), Fondazione Policlinico Universitario ‘Agostino Gemelli’ IRCCS, Rome, Italy; Università Cattolica del Sacro Cuore di Roma, Rome, Italy; General Surgery Unit, Fatebenefratelli Isola Tiberina – Gemelli Isola, Rome, Italy; Gemelli Pancreatic Center, CRMPG (Advanced Pancreatic Research Center), Fondazione Policlinico Universitario ‘Agostino Gemelli’ IRCCS, Rome, Italy; Università Cattolica del Sacro Cuore di Roma, Rome, Italy

Postoperative pancreatic fistula (POPF) is the most concerning post-pancreatoduodenectomy (PD) complication, potentially leading to additional adverse events^1^. Although several surgical and anatomical factors have been recognized as predisposing to POPF^1^, the correlation between postpancreatectomy acute pancreatitis (PPAP) and POPF is still under investigation. Some authors report PPAP as an indirect sign of POPF, suggesting that local inflammation may cause pancreatic oedema and impair the anastomotic healing, contributing to POPF development^2^. Others identify PPAP as an independent complication not necessarily linked to POPF^3^. These discrepancies are partly due to the long-standing absence of a universal definition of PPAP. The International Study Group of Pancreatic Surgery (ISGPS) provided a consensus definition of PPAP based on postoperative hyperamylasaemia (POH) and radiological alterations^4^. This definition shows a more detrimental post-PD course in the case of PPAP onset^5^, but the correlation between PPAP and POPF development is still lacking. This study aimed to analyse the impact of PPAP on the clinical course after PD, focusing on the potential correlation between PPAP and POPF occurrence, and further evaluating the clinical course based on the independent or combined occurrence of these complications.

All clinicodemographic and perioperative data of patients who underwent PD from January 2006 to November 2023 at the Pancreatic Surgery Unit of the Fondazione Policlinico Universitario Agostino Gemelli IRCCS (Istituti di ricovero e cura a carattere scientifico) of Rome were retrospectively retrieved. Postoperative complications were graded according to the Clavien–Dindo classification (*Supplementary reference S1*). POPF, delayed gastric emptying (DGE), postpancreatectomy haemorrhage (PPH) and PPAP were classified according to the ISGPS criteria^4^ (*Supplementary references S2–S4*). POPF monitoring was based on drainage amylase values, while, per protocol, serum amylase values for PPAP diagnosis were evaluated on postoperative day (POD) 1 and 3, more rarely on POD 2. Statistical analysis is reported in the *Supplementary methods*.

During the study interval, 620 patients underwent PD. POH was documented in 74 patients (11.9%) and PPAP in 70 patients (11.3%; 60 grade B and 10 grade C). PPAP patients had higher rates of Clavien–Dindo equal to or greater than grade IIIb complications, including DGE, abscesses, biliary fistula, sepsis, pneumonia and cardiac complications. PPAP was also associated with a more severe POPF, with 35 PPAP patients (50%) developing a clinically relevant (CR)-POPF compared with 98 (17.8%) in the no-PPAP group (*Table S1*). Predictive factors for PPAP included a soft pancreatic texture and a Wirsung diameter equal to or less than 3 mm. Both were confirmed as independent predictors of PPAP (*Table S2*).

Of 133 (21.4%) patients with CR-POPF (105 grade B and 28 grade C), 35 (26.3%) had PPAP while 26 (19.5%) presented POH. Lesions other than pancreatic adenocarcinoma, a soft pancreatic texture, a Wirsung diameter equal to or less than 3 mm and POH were independent predictive factors for CR-POPF (*Table S3*).

*Table 1* shows the post-PD clinical course according to the development of PPAP and CR-POPF alone or in association. Patients who developed both PPAP and CR-POPF presented a significantly higher rate of Clavien–Dindo equal to or greater than grade IIIb complications, PPH and reoperation on as compared with PPAP alone (10 patients *versus* 6, *P* = 0.042; 5 patients *versus* 0, *P* = 0.020; 12 patients *versus* 5, *P* = 0.032 respectively). Patients who presented CR-POPF alone as compared with PPAP alone had a higher frequency of Clavien–Dindo equal to or greater than grade IIIb complications, PPH and reoperation on (35 patients *versus* 6, *P* = 0.041; 10 patients *versus* 0, *P* = 0.050; 38 patients *versus* 5, *P* = 0.008 respectively). No difference was noted in terms of postoperative outcomes between CR-POPF alone and CR-POPF + PPAP.

**Table 1:** Postoperative outcomes stratified for PPAP and/or CR-POPF occurrence

Variables	PPAP+/CR-POPF− (*n* = 35)	PPAP−/CR-POPF+ (*n* = 98)	PPAP+/CR-POPF+ (*n* = 35)	P*	P**	P***
Clavien–Dindo ≥IIIb	6 (17.1)	35 (35.7)	10 (28.6)	**0**.**04**	0.44	**0**.**04**
PPH	0	10 (10.2)	5 (14.3)	**0**.**05**	0.51	**0**.**02**
DGE	17 (48.6)	36 (36.7)	14 (40)	0.22	0.73	0.47
Abscess	26 (74.3)	56 (57.1)	23 (65.7)	0.07	0.37	0.43
Biliary fistula	4 (11.4)	6 (6.1)	3 (8.6)	0.3	0.62	0.69
Sepsis	5 (14.3)	9 (9.2)	7 (20)	0.4	0.09	0.52
Pneumonia	3 (8.6)	23 (23.5)	7 (20)	0.06	0.67	0.17
Cardiological complication	2 (5.7)	7 (7.1)	1 (2.9)	0.77	0.36	0.55
Reoperation on	5 (14.3)	38 (38.8)	12 (34.3)	**0**.**008**	0.63	**0**.**03**
LOS (days), median (i.q.r.)	18 (11–20)	16 (12–21)	19 (12–27)	0.75	0.21	0.14
30-day mortality rate	0	3 (3.1)	2 (5.7)	0.29	0.48	0.15

Values are *n* (%) unless otherwise stated. *P value for PPAP+/CR-POPF− and PPAP-/CR-POPF+; **P value for PPAP−/CR-POPF+ and PPAP+/CR-POPF+; ***P value for PPAP+/CR-POPF− and PPAP+/CR-POPF+. Values in bold are statistically significant. PPAP, postpancreatectomy acute pancreatitis; CR-POPF, clinically relevant postoperative pancreatic fistula; PPH, postpancreatectomy haemorrhage; DGE, delayed gastric emptying; LOS, length of hospital stay; i.q.r., interquartile range.

According to the present findings, PPAP and POPF may be considered two distinct clinical entities. A certain correlation between them is undeniable, especially in relation to the recognition of similar independent risk factors for their occurrence and the key role of POH in predisposing to POPF. Their concomitant occurrence inevitably leads to a more severe post-PD course.

## Supplementary Material

zrae107_Supplementary_Data

## Data Availability

Data that support the findings of this study are available upon reasonable request from the corresponding author.
