# Supplementary material for: Post-pancreatectomy acute pancreatitis and pancreatic fistula after pancreatoduodenectomy: two distinct but potentially correlated clinical entities
Source: BJS Open. 2024 Sep 26;8(5):zrae107. doi: 10.1093/bjsopen/zrae107 (PMC11426162; doi:10.1093/bjsopen/zrae107)
Supplement: zrae107_Supplementary_Data [file zrae107_supplementary_data.docx]

**Post-pancreatectomy acute pancreatitis and pancreatic fistula after pancreaticoduodenectomy: two distinct but potentially correlated clinical entities**

Giuseppe Quero^a,b^ MD, PhD; Claudio Fiorillo^a^ MD; Chiara Lucinato^a^, MD; Flavia Taglioni^a^ , MD; Vito Laterza^a^ MD; Edoardo Panza^a^, MD; Giuseppe Massimiani^a^ MD; Teresa Mezza^b,c^ MD, PhD; Roberta Menghi^a,b^ MD; Ludovica Di Cesare^a^ MD; Beatrice Biffoni^a^ MD; Davide De Sio^a^ MD; Fausto Rosa^a,b^ MD, PhD; Vincenzo Tondolo^d^ MD; Sergio Alfieri^a,b^, MD

^a^ Gemelli Pancreatic Center, CRMPG (Advanced Pancreatic Research Center), Fondazione Policlinico Universitario “Agostino Gemelli” IRCCS, Largo Agostino Gemelli 8, 00168 - Rome (Italy)

^b^ Università Cattolica del Sacro Cuore di Roma, Largo Francesco Vito 1, 00168 - Rome (Italy)

^c^ Pancreas Unit, CEMAD Centro Malattie dell'Apparato Digerente, Medicina Interna e Gastroenterologia, Fondazione Policlinico Universitario “Agostino Gemelli” IRCCS, Largo Agostino Gemelli 8, 00168 - Rome (Italy)

^d^ General Surgery Unit, Fatebenefratelli Isola Tiberina – Gemelli Isola, Via di Ponte Quattro Capi, 39, 00186 – Rome (Italy)

**Corresponding author:**

Claudio Fiorillo, MD

Pancreatic Surgery Unit

Department of Surgery, Fondazione Policlinico Universitario “Agostino Gemelli”, IRCCS

Largo Agostino Gemelli, 8

00168 Rome, Italy

tel.: +39 06 30 15 51 33

fax: +39 06 30 15 65 20

ORCID ID: <https://orcid.org/0000-0001-7681-3567>

E-mail: [claudio.fiorillo@policlinicogemelli.it](mailto:claudio.fiorillo@policlinicogemelli.it)

**Supplementary Materials – Index**

| **Supplementary Methods** |  |
| --- | --- |
| Statistical Analysis | *page 3* |
| **Supplementary Tables** |  |
| Supplementary Table 1 | *page 4-5* |
| Supplementary Table 2 | *page 6-8* |
| Supplementary Table 3 | *page 9-11* |
| **References** | *page 12* |
|  |  |

**Supplementary Methods**

Statistical Analysis

Categorical variables were expressed as number and percentages, while continuous variables as median and quartile rank (QR). The Mann-Withney test, Student’s t-test, chi-squared test and Fisher’s exact test were used for the univariate analysis. Cox regression model was performed in order to identify independent predictors of PPAP and clinically relevant (CR) POPF. Data were reported as Odd Ratio (OR) and 95% confidence interval (CI). Data analysis was conducted using SPSS for Windows, version 25 (SPSS Inc., Chicago, IL, United States).

**Supplementary Tables**

| **Supplementary Table 1. Clinico-demographic characteristics and perioperative outcomes according to PPAP development** | | | |
| --- | --- | --- | --- |
| **Variables** | **No-PPAP group (n:550)** | **PPAP group (n:70)** | **p** |
| ***Clinico-demographic features*** |  |  |  |
| **Sex, n (%)** |  |  |  |
| M | 279 (50.7) | 35 (50) | 0.9 |
| F | 271 (49.3) | 35 (50) |  |
| **Age, median (QR)** | 67 (59-74) | 67 (58-74) | 0.72 |
| **BMI, median (QR)** | 25 (22-28) | 25 (22-27) | 0.95 |
| **ASA score, n (%)** |  |  |  |
| 1-2 | 397 (72.2) | 55 (78.6) | 0.25 |
| 3-4 | 153 (27.8) | 15 (21.4) |  |
| **Diabetes, n (%)** | 88 (16) | 12 (17.1) | 0.18 |
| **NAT, n (%)** | 58 (10.6) | 4 (5.7) | 0.2 |
| **Lesion type, n (%)** |  |  |  |
| PDAC | 333 (60.5) | 35 (50) | 0.09 |
| Other | 217 (39.5) | 35 (50) |  |
|  |  |  |  |
| ***Intraoperative features*** |  |  |  |
| **Operative time, min median (QR)** | 340 (310-375) | 350 (315-415) | 0.59 |
| **Pancreatic texture, n (%)** |  |  |  |
| Firm | 336 (61.1) | 21 (30) | **<0.0001** |
| Soft | 214 (38.9) | 49 (70) |  |
| **Pancreatic duct diameter, n (%)** |  |  |  |
| ≤3 mm | 246 (44.7) | 47 (67.1) | **<0.0001** |
| >3 mm | 304 (55.3) | 23 (32.9) |  |
| **Vascular resection, n (%)** | 59 (10.7) | 11 (15.7) | 0.21 |
| **EBL, mL median (QR)** | 295 (90-401) | 335 (115-425) | 0.76 |
|  |  |  |  |
| ***Postoperative course*** |  |  |  |
| **Clavien-Dindo, n (%)** |  |  |  |
| I-II | 263 (48) | 24 (34.3) | **<0.0001** |
| III-IV | 129 (23.5) | 43 (61.4) |  |
| **Clavien-Dindo ≥3b, n (%)** | 67 (12.2) | 16 (22.9) | **0.01** |
| **Serum amylase, U/l median (QR)** |  |  |  |
| POD 1 | 36 (17-89) | 405 (267-888) | **<0.0001** |
| POD 2-3 | 46 (20-85) | 539 (349-678) | **<0.0001** |
| **DGE, n (%)** | 129 (23.6) | 31 (44.3) | **<0.0001** |
| **POPF, n (%)** | 220 (40) | 59 (84.3) | **<0.0001** |
| **POPF grade, n (%)** |  |  |  |
| BL | 122 (22.2) | 24 (34.3) | **<0.0001** |
| B | 81 (14.7) | 24 (34.3) |  |
| C | 17 (3.1) | 11 (15.7) |  |
| **PPH, n (%)** | 29 (5.3) | 5 (7.1) | 0.52 |
| **Abscess, n (%)** | 153 (27.8) | 49 (70) | **<0.0001** |
| **Biliary fistula, n (%)** | 22 (4) | 7 (10) | **0.02** |
| **Sepsis, n (%)** | 16 (2.9) | 12 (17.1) | **<0.0001** |
| **Penumonia, n (%)** | 42 (7.6) | 10 (14.3) | **0.02** |
| **Cardiac morbidity, n (%)** | 15 (2.7) | 3 (4.3) | **0.03** |
| **Reoperation, n (%)** | 73 (13.6) | 17 (24.3) | **0.02** |
| **LOS, median (QR)** | 13 (10-18) | 18 (12-23) | **0.001** |
| **30-day mortality, n (%)** | 15 (2.7) | 2 (2.9) | 0.95 |

PPAP: post-pancreatectomy acute pancreatitis; BMI: body mass index; ASA: American Society of Anesthesiologists; NAT: neoadjuvant treatment; DGE: delayed gastric emptying; POPF: post-operative pancreatic fistula; PPH: post-pancreatectomy hemorrhage; LOS: length of hospital stay

| **Supplementary Table 2. Prognostic factors analysis for PPAP after PD** | | | | | |
| --- | --- | --- | --- | --- | --- |
| **Variable** | **No PAPP, n (%)**  **(n=550)** | **PAPP, n (%)**  **(n=70)** | ***p*** | **Multivariate analysis  (Logistic regression) odds ratio  [95% Confidence Interval]** | **Multivariate *p* value** |
| **Age*** |  |  |  |  |  |
| ≤65 | 226 (41.1) | 31 (44.3) | 0.6 |  |  |
| >65 | 324 (58.9) | 39 (55.7) |  |  |  |
| **Sex** |  |  |  |  |  |
| Female | 271 (49.3) | 35 (50) | 0.9 |  |  |
| Male | 279 (50.7) | 35 (50) |  |  |  |
| **BMI*** |  |  |  |  |  |
| ≤24 | 275 (50) | 34 (48.6) | 0.82 |  |  |
| >24 | 275 (50) | 36 (51.4) |  |  |  |
| **ASA score** |  |  |  |  |  |
| I-II | 397 (72.2) | 55 (78.6) | 0.25 |  |  |
| III-IV | 153 (27.8) | 15 (21.4) |  |  |  |
| **Preoperative diabetes** |  |  |  |  |  |
| Yes | 88 (16) | 12 (17.1) | 0.91 |  |  |
| No | 462 (84) | 58 (82.9) |  |  |  |
| **NAT** |  |  |  |  |  |
| Yes | 58 (10.5) | 4 (5.7) | 0.2 |  |  |
| No | 492 (89.5) | 66 (94.3) |  |  |  |
| **Type of tumor lesion** |  |  |  |  |  |
| PDAC | 333 (60.5) | 35 (50) | 0.09 |  |  |
| Other | 217 (39.5) | 35 (50) |  |  |  |
|  |  |  |  |  |  |
| **Operative time*** |  |  |  |  |  |
| ≤346 min | 300 (54.5) | 37 (52.8) | 0.47 |  |  |
| >346 min | 250 (45.5) | 33 (47.2) |  |  |  |
| **Vascular resection** |  |  |  |  |  |
| Yes | 59 (10.7) | 11 (15.7) | 0.21 |  |  |
| No | 491 (89.3) | 59 (84.3) |  |  |  |
| **EBL** |  |  |  |  |  |
| ≤225 mL | 328 (59.6) | 38 (54.3) | 0.52 |  |  |
| >225 mL | 222 (40.4) | 32 (45.7) |  |  |  |
| **Harvested lymph nodes*** |  |  |  |  |  |
| ≤20 | 360 (65.4) | 46 (65.7) | 0.85 |  |  |
| >20 | 190 (34.5) | 24 (34.3) |  |  |  |
| **Pancreatic texture** |  |  |  |  |  |
| Firm | 336 (61.1) | 21 (30) | **<0.0001** | 3.3 [1.64-6.6] | **0.001** |
| Soft | 214 (38.9) | 49 (70) |  |  |  |
| **Pancreatic duct diameter** |  |  |  |  |  |
| ≤ 3 mm | 246 (44.7) | 47 (67.1) | **<0.0001** | 2.2 [1.55-3.3] | **0.01** |
| >3 mm | 304 (55.3) | 23 (32.9) |  |  |  |

* The mean values were used as cut-off for the univariate and multivariate analyses

PPAP: post-pancreatectomy acute pancreatitis; PD: pancreaticoduodenectomy; BMI: body mass index; ASA: American Society of Anesthesiologists; NAT: neoadjuvant therapy; PDAC: pancreatic adenocarcinoma; EBL: estimated blood loss

| **Supplementary Table 3. Prognostic factors analysis for CR-POPF after PD** | | | | | |
| --- | --- | --- | --- | --- | --- |
| **Variable** | **No CR-POPF, n (%)**  **(n=487)** | **CR-POPF, n (%)**  **(n=133)** | ***p*** | **Multivariate analysis  (Logistic regression) odds ratio  [95% Confidence Interval]** | **Multivariate *p* value** |
| **Age*** |  |  |  |  |  |
| ≤65 | 208 (42.7) | 49 (36.8) | 0.22 |  |  |
| >65 | 279 (57.3) | 84 (63.2) |  |  |  |
| **Sex** |  |  |  |  |  |
| Female | 243 (49.9) | 63 (47.4) | 0.6 |  |  |
| Male | 244 (50.1) | 70 (52.6) |  |  |  |
| **BMI*** |  |  |  |  |  |
| ≤24 | 243 (49.9) | 66 (49.6) | 0.95 |  |  |
| >24 | 244 (50.1) | 67 (50.4) |  |  |  |
| **ASA score** |  |  |  |  |  |
| I-II | 356 (73.1) | 96 (72.2) | 0.83 |  |  |
| III-IV | 131 (26.9) | 37 (27.8) |  |  |  |
| **Preoperative diabetes** |  |  |  |  |  |
| Yes | 78 (16) | 22 (16.5) | 0.88 |  |  |
| No | 409 (84) | 111 (83.5) |  |  |  |
| **NAT** |  |  |  |  |  |
| Yes | 55 (11.3) | 7 (5.3) | 0.04 |  |  |
| No | 432 (88.7) | 126 (94.7) |  |  |  |
| **Type of tumor lesion** |  |  |  |  |  |
| PDAC | 311 (63.9) | 57 (42.9) | **<0.0001** | 0.47 [0.3-0.72] | **0.001** |
| Other | 176 (36.1) | 76 (57.1) |  |  |  |
|  |  |  |  |  |  |
| **Operative time*** |  |  |  |  |  |
| ≤346 min | 253 (52) | 78 (58.6) | 0.09 |  |  |
| >346 min | 234 (48) | 55 (41.4) |  |  |  |
| **Vascular resection** |  |  |  |  |  |
| Yes | 55 (11.3) | 15 (11.3) | 0.99 |  |  |
| No | 432 (88.7) | 118 (88.7) |  |  |  |
| **EBL** |  |  |  |  |  |
| ≤225 mL | 297 (61) | 80 (60.2) | 0.6 |  |  |
| >225 mL | 190 (39) | 53 (39.8) |  |  |  |
| **Harvested lymph nodes*** |  |  |  |  |  |
| ≤20 | 327 (67.1) | 79 (59.4) | 0.08 |  |  |
| >20 | 160 (32.9) | 54 (40.6) |  |  |  |
| **Pancreatic texture** |  |  |  |  |  |
| Firm | 334 (68.6) | 23 (17.3) | **<0.0001** | 5.7 [3.1-10.5] | **<0.0001** |
| Soft | 153 (31.4) | 110 (82.7) |  |  |  |
| **Pancreatic duct diameter** |  |  |  |  |  |
| ≤ 3 mm | 188 (38.6) | 105 (78.9) | **<0.0001** | 2.28 [1.23-4.21] | **0.008** |
| >3 mm | 299 (61.4) | 28 (21.1) |  |  |  |
| **POH** |  |  |  |  |  |
| Yes | 48 (9.9) | 26 (19.5) | **0.002** | 1.92 [1.03-3.6] | **0.04** |
| No | 439 (90.1) | 107 (80.5) |  |  |  |

* The mean values were used as cut-off for the univariate and multivariate analyses

CR-POPF: clinically relevant post-operative pancreatic fistula; PD: pancreaticoduodenectomy; BMI: body mass index; ASA: American Society of Anesthesiologists; NAT: neoadjuvant therapy; PDAC: pancreatic adenocarcinoma; EBL: estimated blood loss; POH: post-operative hyperamylasemia

**References**

1. Clavien PA, Barkun J, de Oliveira ML, Vauthey JN, Dindo D, Schulick RD, *et al.* The Clavien-Dindo Classification of Surgical Complications. *Ann Surg*. 2009 Aug; **250**: 187–196.
2. Bassi C, Marchegiani G, Dervenis C, Sarr M, Abu Hilal M, Adham M, *et al.* The 2016 update of the International Study Group (ISGPS) definition and grading of postoperative pancreatic fistula: 11 Years After. *Surgery*. 2017 Mar; **161**: 584–591.
3. Wente MN, Bassi C, Dervenis C, Fingerhut A, Gouma DJ, Izbicki JR, *et al.* Delayed gastric emptying (DGE) after pancreatic surgery: A suggested definition by the International Study Group of Pancreatic Surgery (ISGPS). *Surgery*. 2007 Nov; **142**: 761–768.
4. Wente MN, Veit JA, Bassi C, Dervenis C, Fingerhut A, Gouma DJ, *et al.* Postpancreatectomy hemorrhage (PPH)–An International Study Group of Pancreatic Surgery (ISGPS) definition. *Surgery*. 2007 Jul; **142**: 20–25.
